# Viral-Bacterial Co-infections in the Cystic Fibrosis Respiratory Tract

**DOI:** 10.3389/fimmu.2018.03067

**Published:** 2018-12-20

**Authors:** Megan R. Kiedrowski, Jennifer M. Bomberger

**Affiliations:** Department of Microbiology and Molecular Genetics, University of Pittsburgh School of Medicine, Pittsburgh, PA, United States

**Keywords:** cystic fibrosis, coinfection, polymicrobial, respiratory infection, biofilm, chronic disease

## Abstract

A majority of the morbidity and mortality associated with the genetic disease Cystic Fibrosis (CF) is due to lung disease resulting from chronic respiratory infections. The CF airways become chronically colonized with bacteria in childhood, and over time commensal lung microbes are displaced by bacterial pathogens, leading to a decrease in microbial diversity that correlates with declining patient health. Infection with the pathogen *Pseudomonas aeruginosa* is a major predictor of morbidity and mortality in CF, with CF individuals often becoming chronically colonized with *P. aeruginosa* in early adulthood and thereafter having an increased risk of hospitalization. Progression of CF respiratory disease is also influenced by infection with respiratory viruses. Children and adults with CF experience frequent respiratory viral infections with respiratory syncytial virus (RSV), rhinovirus, influenza, parainfluenza, and adenovirus, with RSV and influenza infection linked to the greatest decreases in lung function. Along with directly causing severe respiratory symptoms in CF populations, the impact of respiratory virus infections may be more far-reaching, indirectly promoting bacterial persistence and pathogenesis in the CF respiratory tract. Acquisition of *P. aeruginosa* in CF patients correlates with seasonal respiratory virus infections, and CF patients colonized with *P. aeruginosa* experience increased severe exacerbations and declines in lung function during respiratory viral co-infection. In light of such observations, efforts to better understand the impact of viral-bacterial co-infections in the CF airways have been a focus of clinical and basic research in recent years. This review summarizes what has been learned about the interactions between viruses and bacteria in the CF upper and lower respiratory tract and how co-infections impact the health of individuals with CF.

## Factors Promoting Respiratory Infections in Cystic Fibrosis

Cystic fibrosis (CF) is a lethal genetic disease caused by mutations in the Cystic Fibrosis Transmembrane Conductance Regulator (CFTR) gene (1) that result in dysfunction of the CFTR anion channel (2). To date, close to 2,000 individual mutations in the CFTR gene have been identified (3), and these mutations are further sub-divided into five classes based on how they lead to defective production of CFTR protein, resulting in deficiencies in protein folding, intracellular trafficking, and/or gating reviewed in-depth by Rowntree and Harris (4). While CFTR mutations affect most cell types and all mucosal surfaces in the body, manifesting in different types of disease, respiratory disease remains the most heavily-studied pathology of CF. Chronic respiratory infections and the resulting robust but ineffective inflammatory response, culminating in respiratory failure, are the primary causes of death in CF patients.

In the CF respiratory tract, numerous factors resulting from dysfunctional CFTR combine to create an environment that promotes chronic bacterial and recurring viral infections. A dysfunctional CFTR alters the osmolarity of the airway surface liquid (ASL) layer, resulting in dehydrated ASL, and facilitating the buildup of a thick mucus layer. Diminished ASL hydration and thick mucus at the airway epithelial surface leads to failure of mucociliary clearance in the CF respiratory tract due to collapse of airway cilia, thereby preventing ciliary beat that normally clears debris, and infectious agents from the lungs. This allows microorganisms to repeatedly infect, eliciting robust inflammatory responses dominated by elevated proinflammatory cytokines, and continued accumulation of neutrophils in the CF airway (5). However, these inflammatory responses are ineffective at clearing pathogens in the CF lung, instead creating a hyperinflammatory cycle that leads to host tissue damage, respiratory failure, lung transplant or eventually, death (6). Additionally, the dysregulated conductance of bicarbonate anions by the CFTR channel in CF results in improper mucus formation and an altered ASL pH, which impacts the function of secreted antimicrobial peptides, disrupting a first line of defense against invading bacterial pathogens (7–9). Together, these deficiencies in CF respiratory tract physiology prevent efficient clearance of pathogens from the airways, allowing for the establishment of a robust community of microbes.

## The CF Respiratory Microbiome

### Identification of Bacterial Species in the CF Microbiome

The microbial community in the CF lung is complex, and lung health is affected by the presence and interactions of bacteria, fungi, and respiratory viruses (10, 11). Identification of bacterial species in the CF airways has traditionally relied on culture of bacteria by clinical microbiology laboratories from expectorated sputum samples, respiratory swabs, or samples obtained through bronchoscopy (referred to as culture-dependent methods) (12). Advances in next-generation sequencing have made it possible to identify populations of bacteria residing in the airways without culturing through the isolation of genomic DNA from CF patient samples and sequencing of the gene encoding the bacterial 16S ribosomal subunit. The 16S ribosomal subunit contains variable regions whose sequences can be assigned to bacteria at the species level (termed culture-independent methods) (13). Because culture-dependent methods require knowledge of which bacterial species to target for identification and how to isolate them, known bacterial pathogens were the main species identified from CF respiratory samples prior to culture-independent methods. With the advent of culture-independent methods, it became appreciated that in addition to traditional pathogens, many other bacterial species often associated with the oral cavity or upper respiratory tract, and considered commensal or colonizing organisms were present in the CF lung at high abundance (14). These newly recognized populations included many species of anaerobic bacteria, which previously were not identified, as clinical laboratories did not use culture methods that would allow anaerobic growth (15). It remains a debated issue in the field as to whether all bacterial species identified via culture-independent methods are truly established in the CF lower airways, or if presence of these species is due to contamination of samples by oral or upper respiratory tract microbes during the collection process (11, 16).

### Commensal Microbiome Members

Focusing on bacterial members of the CF microbiome, from culture-independent studies we find that before chronic infections are established by pathogens, the CF lung is colonized by several genera of commensal bacteria. Studies have identified core bacterial airway microbiome members as belonging to the genera *Streptococcus, Prevotella, Veillonella, Rothia, Granulicatella, Gemella*, and *Fusobacterium* (14, 17, 18). Many factors can impact individual patients' respiratory microbiomes, most notably age and antibiotic exposures. Studies of longitudinal samples collected from patients over time have found distinct bacterial community profiles exist for younger vs. older CF patients, with pediatric CF patients possessing a greater abundance of core/commensal bacterial species, and a more diverse bacterial microbiome than CF adults (19). It is thought that these core species are displaced over time as patients age and pathogens are introduced and become established in the airways (10, 18). Multiple studies have found that a higher diversity of species in the lung correlates with better patient outcomes, and decreased diversity correlates with an over-abundance of CF pathogens and declines in patient health (10, 17, 18). Like microbiomes associated with other organ systems in healthy adults, the microbiome in the CF airways has ultimately been found to change minimally upon exposure to antibiotics, exhibiting an altered community structure or decrease in overall abundance during treatment but rebounding to the original community structure after treatment ends (20, 21). Roles for interspecies interactions occurring between commensal and pathogenic bacteria in the CF airways are just beginning to be elucidated. Species of commensal streptococci have been found to have direct protective effects toward the lung by inhibiting pathogens such as *P. aeruginosa* (22–24). We have only recently begun to recognize the complexity of the microbial ecology in the CF airways and how dynamics of microbial communities, and not solely presence of pathogens, can contribute to disease outcomes (25). It is likely that many as yet unknown interspecies interactions exist that impact bacterial populations in the CF airways at a given time, and future studies will pinpoint specific mechanisms mediating bacterial crosstalk that may be targeted to alter the abundance of distinct species.

### Bacterial Respiratory Pathogens in CF

A number of bacterial species have been identified as major respiratory pathogens in CF, including *Staphylococcus aureus, Haemophilus influenzae, P. aeruginosa*, and *Burkholderia* complex (3, 26). *S. aureus* is the most frequently isolated bacterial pathogen in CF pediatric populations, whereas *P. aeruginosa* becomes established as CF patients age (3). *S. aureus* is regarded as a commensal species of the nares and upper respiratory tract but is recognized as a pathogen when identified in other body sites, such as the lower airways, and is cultured from over 70% of CF patients (3). *P. aeruginosa* is the dominant pathogen in end-stage CF lung disease, and chronic infection with *P. aeruginosa* is correlated with more severe reductions in pulmonary function measures (27) and mortality in CF patients. Methicillin-resistant (MRSA) and methicillin-sensitive (MSSA) *S. aureus* and *P. aeruginosa* that display enhanced antibiotic resistance during chronic infection pose significant challenges to treatment efforts (28). Non-traditional bacterial pathogens, including *Achromobacter xylosoxidans, Stenotrophomonas maltophilia*, and non-tuberculosis Mycobacterium (NTM), also contribute to respiratory infections in CF patients and have been associated with worsening lung function (26, 29). A mechanism common to most CF bacterial pathogens for evasion of host immune defenses in the CF lung, despite its hyperinflammatory state, is growth in bacterial aggregates or biofilms. During the transition from acute to chronic infection, *P. aeruginosa, S. aureus*, and other bacterial pathogens exhibit altered metabolism, decreased growth rate and up-regulated expression of antibiotic resistance genes, and these changes, together with increased production of polymeric matrix materials, protect organisms within biofilms from the hostile environment in the CF lung (30–32).

## Respiratory Viruses in CF

### Acute Respiratory Viral Infections in CF

Pediatric and adult CF patients experience frequent acute respiratory virus infections. Specific respiratory viruses responsible for infections are identified when patients present with symptoms indicative of a viral infection, leading physicians to take a viral swab from which genetic material is extracted for PCR, and a viral panel is performed consisting of primer sets specific to common viral culprits. The true incidence of viral infections is likely under-reported for several reasons, including infrequent use of viral swabs and incomplete PCR panels to detect viral infections, as well as the fact that not all patients present with symptoms during a viral infection (33, 34). The most commonly identified viral pathogens in CF populations are respiratory syncytial virus (RSV), human rhinovirus (RV), Influenza types A and B, and parainfluenza, all belonging to families of RNA viruses (34–36). It has been reported that close to 40% of children with CF are hospitalized at some point for severe respiratory infections, and of these hospitalizations, respiratory viruses were identified in 50% of patients, with RSV predominating (33). While in non-CF populations RSV is thought to be almost exclusively a pediatric pathogen, RSV infections are frequent in both adult and pediatric CF patients, and can result in severe symptoms. RSV infection may result in upper respiratory disease, including rhinitis, cough, fever, and acute otitis media, or progress to the lower respiratory tract, resulting in bronchiolitis or pneumonia in children, and exacerbate existing chronic airway disease in adults (37). RSV infection is especially aggressive in young infants with CF, leading to significant respiratory morbidity (38).

### Links Between Viral Infections and Exacerbations

CF patients frequently experience periods of rapidly worsening respiratory symptoms, termed pulmonary exacerbations (39). Pulmonary exacerbations are typically defined by a decrease in lung function or increases in patient symptoms, however symptoms and severity of exacerbations vary from patient to patient and can be triggered by a multitude of causes (40). Exacerbations are often treated by initiating courses of additional antibiotics, increasing airway clearance therapies, or hospitalization in severe cases (41). Many clinical studies have now linked viral infections with pulmonary exacerbations (33, 35, 36, 42). Respiratory viral infections account for at least 40% of pulmonary exacerbations of CF adults (38, 43) and are linked to pulmonary function decline, antibiotic use, prolonged hospitalizations, and increased respiratory symptoms in CF patients (44–46). Respiratory viruses most frequently cultured during periods of exacerbation include the major viral pathogens appreciated in CF: influenza A and B, RSV, and RV (47–50).

### Severity of Viral Infections in CF

CF patients are known to be pre-disposed to chronic bacterial infections, and several groups have examined whether CF disease also leads to more severe respiratory viral infections. *In vitro* studies evaluating CF vs. non-CF primary human bronchial epithelial cells in culture found that RV replication was increased in CF cells (51). Enhancement of viral infection could be attributed to a diminished innate antiviral response in CF cells, which showed weaker induction of interferon and expression of some interferon-stimulated genes, as compared to non-CF controls (52). A clinical study evaluating severity of RV infections in CF children compared to non-CF pediatric patients with asthma, non-CF bronchiectasis or healthy controls found CF patients had a higher prevalence of RV, and higher viral load in bronchoalveolar lavage (BAL), both when patients were stable and at even higher levels during pulmonary exacerbations (53). Higher RV load correlated with worse lung function scores in CF children, and RV infection in CF resulted in lower levels of inflammatory markers than in non-CF children, again indicating a dysregulated innate immune response in CF patients could be responsible for increased severity of viral infections (53). A longitudinal study reported that RV was identified more frequently in CF children than non-CF subjects, and RV infections in CF children persisted longer (54). These studies suggest inherent properties of CF airway cells may make the CF airway epithelium more prone to viral infection, and together with what is known regarding links between airway physiology and bacterial respiratory infection in CF, these factors could have important implications in cases of viral-bacterial co-infections.

## Viral-Bacterial Co-Infections in CF

**Figure 1:**
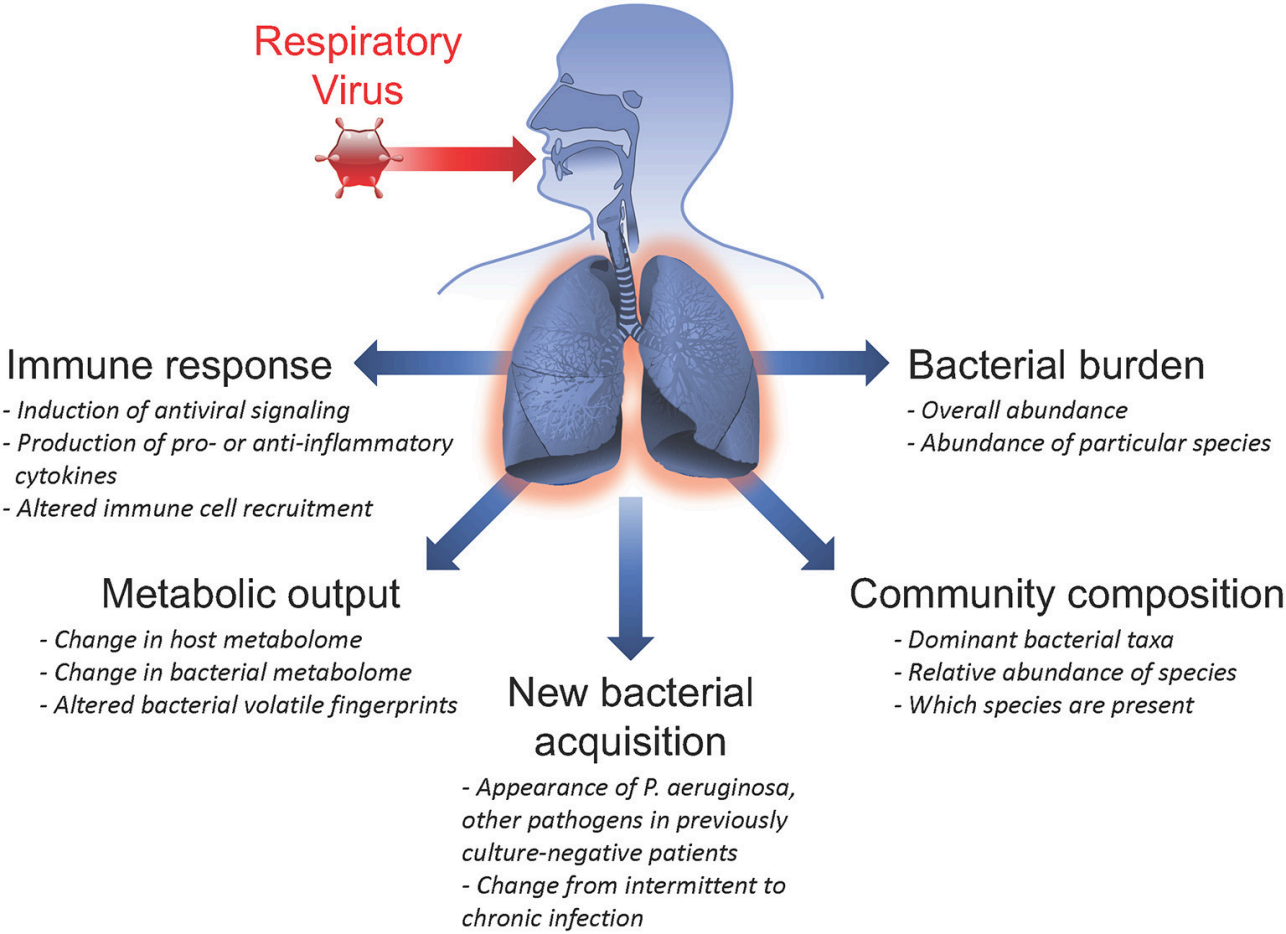
Summary of respiratory virus infection outcomes impacting viral-bacterial interactions in Cystic Fibrosis. Acute respiratory viral infections are known to affect subsequent infection with bacterial pathogens and influence pre-existing chronic bacterial infections in individuals with Cystic Fibrosis. Some ways in which respiratory viruses have been shown to impact bacterial infections include: inducing the host immune response; altering metabolic output of both host and infecting bacteria; causing new bacterial acquisition in patients who were previously culture-negative; altering bacterial community composition by shifting relative abundance of specific bacterial species; and inducing fluctuations in total bacterial burden.

CF patients are commonly chronically infected with bacterial pathogens and maintain a high abundance of microbes in the respiratory tract, including pathogens, and commensal organisms. These same patient populations also experience frequent acute respiratory viral infections. There are numerous ways in which infection with a viral pathogen can alter the host response, impacting previously existing chronic bacterial infections and microbial communities, potentiating secondary bacterial infections, and/or permitting the acquisition of new bacterial species in the airways (summarized in Figure 1). In this section, we evaluate insights from clinical studies of CF patient populations and mechanistic *in vitro* studies that inform us of viral-bacterial interactions occurring in CF during co-infections.

### Impact of Virus Infection on the CF Microbiome

Temporal changes in microbiome composition could result from a variety of disturbances that alter the environment in the CF airways, including initiation of antimicrobial therapies, mechanical or airway clearance treatments, or an altered host response. Respiratory viral infections can promote the onset of respiratory symptoms, as well as trigger the innate antiviral response in the CF airway epithelium, resulting in induction of antiviral signaling, and inflammation. From non-CF studies, respiratory viral infection is known to skew the immune status of the respiratory tract to be predisposed to secondary bacterial infection, which has been most studied for influenza (55). The altered host immune status following viral infection reduces antibacterial effector functions, like phagocyte recruitment (56), antimicrobial peptide production (57, 58), and protective adaptive immune responses (59–61), increasing susceptibility to bacterial infections. Specifically, interferon-mediated antiviral responses following influenza infection in mice (62) and vaccination with live attenuated influenza in humans (63) have been shown to shift the composition of the upper respiratory microbiome and increase the potential for emergence of *S. aureus* infections. An altered immune status resulting from respiratory viral infection likely also alters the microbial composition of the CF airways, potentially leading to shifts in bacterial populations comprising the microbiome and promoting infection by specific bacterial pathogens.

How respiratory viral infections impact the CF airway microbiome can be evaluated by observing changes in overall bacterial burden (or bacterial load), community composition, or dominant taxa. In non-viral-associated pulmonary exacerbations, the overall bacterial burden in the CF airways rarely changes in the time leading up to an exacerbation or during exacerbations (64–66). Studies evaluating bacterial burden during acute respiratory viral-associated pulmonary exacerbations have produced conflicting results. In one prospective study of CF adults, *P. aeruginosa* density in sputum was not found to increase during exacerbation, compared to patients' stable states in either the presence or absence of a respiratory virus co-infection (67). A similar study design by another group found the opposite to be true: in adult CF patients evaluated, a significantly higher load of *P. aeruginosa* was observed during respiratory-virus associated exacerbations (48). An observational study of CF children found *P. aeruginosa* density was not significantly different between patients experiencing viral- or non-viral-associated exacerbations (47). Additional prospective studies with larger patient cohorts are needed to gain a more definitive understanding of the effects of virus co-infection on overall bacterial burden and burden of specific organisms, like *P. aeruginosa*, in pediatric and adult CF populations.

While the relationship between respiratory virus co-infection and bacterial burden remains unclear, more efforts have been made toward investigating associations between viral infection and culture of specific bacterial pathogens from the CF airways. Findings from multiple studies indicate approximately 15–25% of CF patients undergoing a respiratory viral infection also culture positive for a known CF bacterial pathogen (68–70). Previously uncolonized CF patients have been reported to undergo new acquisition of *P. aeruginosa* following seasonal respiratory virus infections (44). Regarding roles for specific respiratory viruses, both RSV and RV are linked in clinical studies to development of *P. aeruginosa* co-infections and conversion from intermittent to chronic *P. aeruginosa* colonization in CF patients (33, 35, 43, 44, 71). Similarly, other known bacterial respiratory pathogens, including *H. influenzae, Moraxella catarrhalis*, and *Streptococcus pneumoniae*, were cultured more frequently from CF patients experiencing a RV co-infection (68). A separate pediatric study also found that in a cohort of CF children experiencing viral-bacterial co-infections, RV and *S. aureus* were co-cultured more frequently than any other viral-bacterial pair (69). Together, these studies present strong clinical evidence for association of respiratory viral co-infection with presence of bacterial pathogens in the airways and suggest associations between specific species of viruses and bacteria co-cultured that may vary with age.

### Toward Mechanisms Underlying Viral-Bacterial Interactions

Mechanistic studies evaluating the outcomes of viral-bacterial co-infections have been made possible through the availability of isogenic immortalized CF airway epithelial cell lines (72) and access to well-differentiated primary airway epithelial cells cultured from CF lung tissue following lung transplant (73). In cell culture experiments by our group and others, simultaneous inoculation of CF and non-CF airway cells with RSV and *P. aeruginosa* increased adherence of both mucoid and non-mucoid *P. aeruginosa* strains (74, 75). This effect could be blocked by pre-treating non-polarized airway cells with heparin prior to inoculation with RSV and *P. aeruginosa* (75). A recent study from our group found no significant difference in *P. aeruginosa* adherence to polarized CF airway cells with a preceding RSV infection (24–72 h) compared to control CF cells; however, co-infection with RSV, RV, and adenovirus each promoted the growth of *P. aeruginosa* biofilms (74). Treatment of CF airway cells with exogenous type I or type III interferon prior to *P. aeruginosa* inoculation also stimulated biofilm growth, suggesting *P. aeruginosa* benefits from the innate antiviral response in CF airway cells. Together, these studies suggest physical binding of RSV to *P. aeruginosa* or the CF airway cell surface may facilitate initial adherence of *P. aeruginosa* to the epithelium, whereas progression of viral infections and activation of interferon-stimulated innate antiviral signaling pathways may play a role in promoting chronic *P. aeruginosa* growth in biofilms. New work from our group finds co-infection with RSV and RV also promotes *S. aureus* biofilm growth on CF airway cells through as yet unidentified mechanisms (76).

Bacteria depend largely on nutrients and metabolites supplied by the host during infection, and virus co-infection has been shown to alter nutritional availability, thereby influencing bacterial responses. Iron is known to be a key nutrient required for growth and pathogenesis of many pathogens (see the Frontiers Research Topic, “Role of Iron in Bacterial Pathogenesis”). The host normally sequesters iron and other essential metals from invading microbes through a process termed nutritional immunity (77), but these mechanisms have been found to be dysregulated during virus infection. In CF airway cell studies, it was discovered that RSV infection promoted increased secretion of iron-bound host transferrin protein, which stimulated *P. aeruginosa* biofilm growth (74). Lipocalin-2, a host antimicrobial protein that sequesters iron (78), was found to be reduced during influenza A infection through virus-mediated suppression of NF-kB activation and IL-1B expression, exacerbating *S. aureus* acute pneumonia in mice (58). Other potential nutrient sources in the airways, such as surfactant proteins (79–81), and mucins (82–84), are known to change during virus co-infections, and there is evidence that CF bacterial pathogens can utilize these nutrient sources (85–88), but specific links between these nutrient shifts and viral-bacterial co-infections CF have not yet been confirmed.

Conversely, bacterial interactions with the CF airway epithelium can also alter subsequent virus infection. In one study, pre-infection of CF human bronchial cells with *P. aeruginosa* was found to diminish the interferon response to RV infection and resulted in a higher RV load than RV infection alone (89). As CF cells showed increased generation of reactive oxygen species (ROS) at baseline compared to non-CF cells, treatment of CF cells with antioxidants prior to *P. aeruginosa* infection helped to restore the IFN response, and it was observed that while RV infection alone acted through PI-3 kinase to induce Akt phosphorylation, this was prevented by pre-infection of CF airway cells with *P. aeruginosa*. A later study evaluating the effects of *P. aeruginosa* secreted factors on primary CF and non-CF airway cells observed no effect of pre-treatment on RV load or antiviral gene expression; however, pre-exposure of cells to *P. aeruginosa* secreted factors did potentiate IL-8 production upon subsequent RV infection (90). Another secreted *P. aeruginosa* protein, Cif, also potentiated virus infections in CF airway epithelial cells by preventing MHC class I antigen presentation and CD8 T cell-mediated clearance of influenza A-infected cells (91). Taken together, these findings suggest a complex interplay between bacterial pathogens, respiratory viruses, and the innate immune response in the CF airway epithelium, where an appropriate immune response to one pathogen may alter secondary infection by another pathogen. The effects of virus co-infection on many prominent CF bacterial pathogens has yet to be evaluated, and relationships between respiratory viruses and bacteria in multi-species polymicrobial infections representative of the complex communities existing in the CF airways is also an underexplored area.

## Impact of Therapeutics on Co-Infections

### Antimicrobial Treatments for Viral and Bacterial Infections

As we've observed with the host immune response, attempts to clear one type of pathogen may have unintended effects on other microbes in the CF airways. The same may be true for cases of antiviral or antibacterial treatments administered to CF patients. It is appreciated that despite intense antibiotic therapy and even with alternating antibiotic courses, chronic infections with bacterial pathogens established as biofilms in the CF airways resist clearance through multiple mechanisms reviewed in Høiby et al. (92) and Lambert (93). Several therapies are now shown to impact both viral and bacterial pathogens, potentially leading to new therapeutic options for polymicrobial infections. We recently reported that an engineered antimicrobial peptide therapy, WLBU2, reduced both bacterial biofilm, and RSV titers in a mixed infection model *in vitro* (94). In addition, members of the macrolide class of antibiotics, including erythromycin, azithromycin, and bafilomycin, which are known to effect antibacterial activity by binding to bacterial ribosomal subunits to inhibit protein synthesis (95) were also found to have anti-inflammatory effects. By blocking production of the pro-inflammatory cytokines IL-6 and IL-8, macrolide antibiotics reduced neutrophil recruitment to sites of injury, and infection (96). In non-CF bronchial epithelial cells, azithromycin (97), bafilomycin (98), and clarithromycin (99) treatments were found to reduce RV replication by increasing induction of interferon-stimulated antiviral genes, demonstrating that in addition to its anti-inflammatory, and anti-bacterial properties, azithromycin has anti-viral activity. In CF airway cells, azithromycin also reduced RV replication and increased RV-induced expression of interferon and interferon-stimulated antiviral genes; however, azithromycin did not prevent induction of IL-6 or IL-8 during RV infection, suggesting that the anti-inflammatory effects of azithromycin are diminished during a virus infection (51). Administration of azithromycin as an antiviral or anti-inflammatory agent could provide a potential therapeutic option for CF patients, yet as macrolide resistance is known to be widespread in clinical isolates from chronic airway infections (100), it is important to keep in mind the broader effects antibiotic administration may have on the CF microbiome.

As viral infections can lead to severe respiratory morbidity and are linked to exacerbations in CF populations, there is a demand for effective anti-viral therapies, especially for major CF pathogens like RV and RSV for which no successful vaccine exists. RSV immunotherapy was shown to be effective at preventing lower respiratory tract infections and reducing symptom severity in high-risk infants, and young children (101). A humanized monocolonal antibody treatment for RSV, palivizumab, was developed (102) and prophylactic treatment with palivizumab significantly reduced hospitalizations (103), and incidences of respiratory-related illness (104) in CF children compared to untreated control groups. While potentially effective at preventing RSV infection, palivizumab prophylaxis is costly and has shown limited benefits for populations that do not regularly have high incidence of RSV-related hospitalizations (105), leading some to propose that anti-RSV therapy would be best-reserved for treatment during infections, not as prophylaxis, or for fall, and winter seasons when probability of virus-related illnesses and hospitalizations typically increases (106). As it has been observed that virus co-infection promotes *P. aeruginosa* colonization, a secondary benefit of antiviral therapies could be a delay in acquisition of bacterial pathogens in CF children. However, a recent study found that prophylactic treatment of CF infants with palivizumab to prevent RSV infection did not delay acquisition of either *P. aeruginosa* or *S. aureus* (107). A separate retrospective study found that although palivizumab reduced RSV-related hospitalizations and overall *P. aeruginosa* chronic colonization rates did not differ between treatment and control groups, the time to first *P. aeruginosa* isolate was significantly earlier in palivizumab-treated CF children (108). Many factors could have affected these outcomes, including patients' genetics, environmental exposures, and differences in clinical care quality and access. Broader studies evaluating the impact of palivizumab on the CF microbiome, including changes in abundance of commensal and pathogenic bacterial species, could shed light on how anti-viral therapies affect viral-bacterial-host interactions.

### CFTR Modulators and Impact on Infections

In the field of CF research and patient care, there is great excitement surrounding the promise of CFTR modulating drugs that improve mutated CFTR rescue to the cell surface (correctors) or modulate activity of dysfunctional CFTR protein channels (potentiators). Three drugs have undergone clinical trials and are now options for CF patients with specific CFTR mutations: the potentiator ivacaftor and correctors lumacaftor and tezacaftor (109, 110). Trials showed CF patients treated with CFTR modulators had improved lung function and decreased rates of pulmonary exacerbations, hospitalization, and IV antibiotic use, and the first studies on how these treatments impact respiratory microbiology in CF patients are now becoming available. During clinical trials, CF patients receiving ivacaftor, and lumacaftor were still found to experience adverse events, including upper respiratory infections (usually attributed to acute respiratory viral infection), at rates similar to placebo groups (111–113), and CF patients experiencing acute upper or lower respiratory infections have also been excluded from trials on the basis that this could confound results, although this practice may limit knowledge of the impact of these drugs during infection (113).

*In vitro* studies of ivacaftor, whose structure resembles that of quinolone antibiotics, found that ivacaftor has dose-dependent antibacterial activity against *S. aureus* and *S. pneumoniae* clinical isolates, and synergy of ivacaftor and the anti-Gram positive antibiotic vancomycin was observed (114). Ivacaftor was also observed to have a milder antimicrobial effect toward *P. aeruginosa* that was improved in combination with the anti-pseudomonal antibiotic ciprofloxacin. These results suggest that in addition to the intended ability of CFTR modulators to improve CFTR production and function, these treatments could have the added benefit of helping to reduce certain bacterial populations in the CF airways.

Studies evaluating changes in the airway microbiome of CF patients undergoing CFTR modulator therapies can begin to inform us if the above observed properties of CFTR modulators translate to the clinic. A small study using quantitative PCR and 16S sequencing to evaluate the microbiome of three CF children undergoing ivacaftor treatment found no significance in overall bacterial burden or bacterial genera represented prior to and following treatment, and individual patients' microbiomes pre- and post-treatment were found to be more similar than treated vs. non-treated microbiomes across all patients (115). Recent work evaluating CF adults showed that ivacaftor treatment reduced *P. aeruginosa* and overall bacterial density in sputum samples, yet despite immediate effects, the same clonal isolates of *P. aeruginosa* sampled prior to treatment persisted in the airways of CF patients after ivacaftor (116). Inflammatory markers in patient sputum were found to be decreased in patients through mass spectrometry analysis of sputum proteins, and a separate study unexpectedly found that ivacaftor treatment dampened the interferon-gamma response and impaired monocyte recruitment, effects that modulate immune responses in the respiratory tract and could potentially influence disease outcome (117). Trials conducted in adult populations, many of whom are already chronically colonized with *P. aeruginosa*, do not allow for evaluation of the potential impact of CFTR modulators in preventing *P. aeruginosa* acquisition. Initiating new studies in younger patient populations following modulator treatment and following patients that begin treatment culturing *P. aeruginosa*-negative for longer periods of time post-treatment will be important to address the question of whether CFTR modulators impact *P. aeruginosa* acquisition. No specific effects of CFTR modulators on viral-bacterial co-infections have been reported to date, and valuable information could be gained from testing for the presence of specific respiratory viruses in patients undergoing modulator therapy, along with measuring changes in bacterial microbiome constituents. The above described effects of CFTR modulators on specific bacterial pathogens and the host immune response suggest co-infections will likely be impacted by such therapies and warrant further study.

## New Techniques for Evaluation of Viral-Bacterial Co-Infections

Traditional means of diagnosing viral and bacterial infections through specific PCR panels or culture-based techniques, respectively, have provided the majority of our current knowledge regarding which microbes comprise the CF respiratory microbiome and can be considered pathogens in the CF airways. However, as previously mentioned, these methods of identification are limited, as each requires prior knowledge of which viruses to screen for or which bacteria to culture, and therefore we may be underestimating the number of species that exist in the airway environment and the impact they have on CF respiratory disease. The recent trend toward 16S studies has identified additional members of the bacterial microbial community, and the application of new techniques such as metagenomics, metatranscriptomics, and metabolomics to the study of the CF microbiome may begin to reveal previously unknown roles for new microbial species and offer unprecedented insight into their functions in the CF respiratory tract.

### Metagenomics and Metatranscriptomics

Metagenomic studies evaluating the total genomic content of samples have great potential to identify a broader range of viruses and other microbes in CF, in addition to bacterial species. Such evaluations are technically challenging from a computational standpoint, and to date, few metagenomic studies have been published applying this technique to the study of viromes in CF populations. While RNA viruses have been the most appreciated viruses in causing acute respiratory infections in CF, a metagenomic study evaluating DNA viruses identified genomes of herpesviruses, and retroviruses in CF sputum and found overall eukaryotic viral diversity was low in both CF and non-CF individuals (118). The majority of viral diversity in airway microbial communities was found to be derived from populations of bacteriophage, with CF phage communities being more similar to one another than non-CF phage, and indicative of the dominant bacterial species residing in the CF airways that comprise the host range of the phage (118, 119). Although phage are not traditionally thought of as viruses that impact human health, recent work has shown that phage affect *P. aeruginosa* biofilm assembly and promote survival of bacteria in biofilms by enhancing adhesion and tolerance to antibiotics (120), and the role of phage in promoting a healthy microbiome in other organ systems, namely the gastrointestinal tract, has been a rapidly growing area of research outside of CF (121, 122). Going further, functional genomic analyses that consider the predicted functions of all genes present in the total DNA from a sample predicted the viromes of CF patients had a separate set of core metabolic functions compared to healthy subjects (118), with enrichment of genes for metabolizing aromatic amino acids. This suggests the host organisms of these phage have a specialized metabolism specific to CF disease, and the genes carried by phage represent factors necessary for survival in the CF airways.

Similar to metagenomics, metatranscriptomics is the analysis of the total RNA content of a sample and can thus account for changes in expression of host and microbial genes. To date, limited metatranscriptomics studies have been performed evaluating both host and pathogen gene expression in the same sample. In a mouse model of acute *P. aeruginosa* infection, metatranscriptomics revealed genes related to *P. aeruginosa* outer membrane vesicle production, and iron uptake and utilization were significantly upregulated, indicating the importance of iron-mediated regulation and scavenging *in vivo* (123). In infected mice, expression of pro-inflammatory cytokines and chemokines associated with toll-like receptor signaling were induced. Recent dual-RNA sequencing experiments from our group show polarized CF airway cells and *S. aureus* exhibit altered transcriptional profiles during co-culture in the presence of RSV co-infection (76). In CF cells, differences in innate immune and inflammatory signaling were observed in the presence of RSV, *S. aureus* or viral-bacterial co-infection, and *S. aureus* exhibited an altered metabolic transcriptional profile during virus co-infection, with upregulation of genes for cofactor biosynthesis and amino acid utilization, perhaps reflecting altered availability of protein substrates in the airway surface liquid of virus-infected CF cells. The use of next-generation sequencing approaches is expanding, and these techniques as applied to CF in future studies will no doubt expand our view of viruses and other underappreciated microbes that exist in the CF airways.

### Metabolomics

Supplementing metagenomic studies that identify which microbial species are present in the airways, metabolomics studies can account for microbial and host-derived metabolites and proteins. For studies of biological samples, liquid chromatography-mass spectrometry (LC-MS) is often used to identify small molecules, and liquid chromatography-tandem mass spectrometry (LC-MS/MS) can be applied to identify sequences of individual peptides for proteomic analysis in complex samples (124).

A novel application of MS techniques is analysis of volatile compounds found in breath to diagnose infections. Volatile compounds produced during microbial metabolism can provide species-specific signatures based on knowledge of individual species' metabolic capabilities from genome sequences, and variation in volatile compound production can be an indicator of compounds available for use in the environment. Early studies in CF utilized the knowledge that *P. aeruginosa* produces cyanide, and using cyanide as a biomarker, selected ion flow mass spectrometry (SIFT-MS) was able to identify the presence of *P. aeruginosa* in the airways by sampling the breath of infected CF patients (125, 126). Later studies showed that beyond identifying presence of a specific molecule, groups of *P. aeruginosa*-colonized CF patients could be differentiated from non-colonized CF patients based on overall volatile breath profiles (127). Using genomic data available for other CF-related bacterial species to predict organisms capable of producing specific volatile compounds, high levels of acetaldehyde, ethanol, and methanol in CF subjects' breath were linked to *Lactococcus, Escherichia* and *Rothia* species, respectively (128), confirming that metabolic predictions based on genetic sequence could translate to positive identification in patient samples for species other than *P. aeruginosa*.

Translating breath detection to bench studies, a volatile fingerprint could be identified for CF airway cells co-cultured with *P. aeruginosa* (129). Evaluating RSV-*P. aeruginosa* co-infections in CF airway cells, different levels of volatile compounds were found to be produced during co-infection rather than infection with either *P. aeruginosa* or RSV alone, and predictive models were able to discriminate *P. aeruginosa*-infected cells, but not cells undergoing only RSV infection (129). Breath diagnosis could prove to be a quick, non-invasive, and culture-independent means for diagnosing the presence of CF pathogens, and with additional knowledge of the links between overall metabolic state and respiratory function, breath testing could serve as an indicator of a stable or exacerbating CF airway environment. The prospect of using breath analysis to identify a virus co-infection is intriguing, and *in vitro* studies require further investigation and translation into CF patients undergoing respiratory viral infections to confirm specific volatile signatures for co-infections.

In summary, the individual roles of bacterial and viral infections in CF respiratory disease have long been appreciated, and independently, viral infections, and chronic bacterial infections are known to influence pulmonary exacerbations and progression of respiratory function decline. Now, advances in sequencing technology have facilitated our understanding of the complexity of the microbial communities in the CF airways, bringing to light new interactions between bacteria and viruses in the airways that suggest microbial population dynamics and interplay between microbes and the host, and not just the presence of known pathogens, could be the true drivers of CF respiratory disease. While metabolomics has not yet been used to evaluate differences in metabolite profiles in CF patients with respiratory viral infections or viral-bacterial co-infections, the technology used in the above described studies provide an exciting window into potential host- and bacterial-associated changes in metabolism that likely accompany co-infections. Expanding the use of such new technologies in clinical and basic CF research will undoubtedly allow us to better understand these microbe-microbe and microbe-host interactions, improving our ability to more accurately diagnose and treat respiratory infections in CF patients and informing us of underlying mechanisms of microbial pathogenesis in the CF respiratory tract.

## Author Contributions

MK and JB conceptualized and wrote the manuscript.

### Conflict of interest statement

The authors declare that the research was conducted in the absence of any commercial or financial relationships that could be construed as a potential conflict of interest.

## References

[1] Rommens JM Iannuzzi MC Kerem B Drumm ML Melmer G Dean M. Identification of the cystic fibrosis gene: chromosome walking and jumping. Science (1989) 245:1059–65. 10.1126/science.2772657 2772657

[2] Welsh MJ Smith AE. Molecular mechanisms of CFTR chloride channel dysfunction in cystic fibrosis. Cell (1993) 73:1251–4. 10.1016/0092-8674(93)90353-R 7686820

[3] Cystic Fibrosis Foundation 2015 CFF Patient Registry Annual Data Report. (2016). 1–94.

[4] Rowntree RK Harris A. The phenotypic consequences of CFTR mutations. Ann Hum Genet. (2003) 67:471–85. 10.1046/j.1469-1809.2003.00028.x 12940920

[5] Nichols D Chmiel J Berger M. Chronic inflammation in the cystic fibrosis lung: alterations in inter- and intracellular signaling. Clin Rev Allergy Immunol. (2008) 34:146–62. 10.1007/s12016-007-8039-9 17960347

[6] Gifford AM Chalmers JD. The role of neutrophils in cystic fibrosis. Curr Opin Hematol. (2014) 21:16–22. 10.1097/MOH.0000000000000009 24253427

[7] Pezzulo AA Tang XX Hoegger MJ Alaiwa MHA Ramachandran S Moninger TO. Reduced airway surface pH impairs bacterial killing in the porcine cystic fibrosis lung. Nature (2012) 487:109–13. 10.1038/nature11130 22763554 PMC3390761

[8] Quinton PM. Role of epithelial HCO3? transport in mucin secretion: lessons from cystic fibrosis. Am J Physiol Cell Physiol. (2010) 299:C1222–33. 10.1152/ajpcell.00362.2010 20926781 PMC3006319

[9] Singh PK Tack BF McCray PB Welsh MJ. Synergistic and additive killing by antimicrobial factors found in human airway surface liquid. Am J Physiol Lung Cell Mol Physiol. (2000) 279:L799–805. 10.1152/ajplung.2000.279.5.L799 11053013

[10] Nguyen LDN Viscogliosi E Delhaes L. The lung mycobiome: an emerging field of the human respiratory microbiome. Front Microbiol. (2015) 6:89. 10.3389/fmicb.2015.00089 25762987 PMC4327734

[11] Surette MG. The cystic fibrosis lung microbiome. Ann Am Thorac Soc. (2014) 11(Suppl. 1):S61–5. 10.1513/AnnalsATS.201306-159MG 24437409

[12] Recommendations of the Clinical Subcommittee of the Medical/Scientific Advisory Committee of the Canadian Cystic Fibrosis Foundation Microbiological processing of respiratory specimens from patients with cystic fibrosis. Can J Infect Dis. (1993) 4:166–9. 10.1155/1993/989086 22346442 PMC3250786

[13] Muyzer G de Waal EC Uitterlinden AG. Profiling of complex microbial populations by denaturing gradient gel electrophoresis analysis of polymerase chain reaction-amplified genes coding for 16S rRNA. Appli Environ Microbiol. (1993) 59:695–700. 7683183 10.1128/aem.59.3.695-700.1993 PMC202176

[14] Mahboubi MA Carmody LA Foster BK Kalikin LM VanDevanter DR LiPuma JJ. Culture-based and culture-independent bacteriologic analysis of cystic fibrosis respiratory specimens. J Clin Microbiol. (2016) 54:613–9. 10.1128/JCM.02299-15 26699705 PMC4767965

[15] Sibley CD Grinwis ME Field TR Eshaghurshan CS Faria MM Dowd SE. Culture enriched molecular profiling of the cystic fibrosis airway microbiome. PLoS ONE (2011) 6:e22702. 10.1371/journal.pone.0022702 21829484 PMC3145661

[16] Zemanick ET Sagel SD Harris JK. The airway microbiome in cystic fibrosis and implications for treatment. Curr Opin Pediatr. (2011) 23:319–24. 10.1097/MOP.0b013e32834604f2 21494150

[17] Coburn B Wang PW Diaz Caballero J Clark ST Brahma V Donaldson S. Lung microbiota across age and disease stage in cystic fibrosis. Sci Rep. (2015) 5:10241. 10.1038/srep10241 25974282 PMC4431465

[18] Filkins LM Hampton TH Gifford AH Gross MJ Hogan DA Sogin ML. Prevalence of streptococci and increased polymicrobial diversity associated with cystic fibrosis patient stability. J Bacteriol. (2012) 194:4709–17. 10.1128/JB.00566-12 22753064 PMC3415522

[19] Cox MJ Allgaier M Taylor B Baek MS Huang YJ Daly RA. Airway microbiota and pathogen abundance in age-stratified cystic fibrosis patients. PLoS ONE (2010) 5:e11044. 10.1371/journal.pone.0011044 20585638 PMC2890402

[20] Fodor AA Klem ER Gilpin DF Elborn JS Boucher RC Tunney MM. The adult cystic fibrosis airway microbiota is stable over time and infection type, and highly resilient to antibiotic treatment of exacerbations. PLoS ONE (2012) 7:e45001. 10.1371/journal.pone.0045001 23049765 PMC3458854

[21] Smith DJ Badrick AC Zakrzewski M Krause L Bell SC Anderson GJ. Pyrosequencing reveals transient cystic fibrosis lung microbiome changes with intravenous antibiotics. Eur Respir J. (2014) 44:922–30. 10.1183/09031936.00203013 25034564

[22] Scoffield JA Wu H. Oral streptococci and nitrite-mediated interference of *Pseudomonas aeruginosa*. Infect Immun. (2015) 83:101–7. 10.1128/IAI.02396-14 25312949 PMC4288860

[23] Scoffield JA Wu H. Nitrite reductase is critical for *Pseudomonas aeruginosa* survival during co-infection with the oral commensal *Streptococcus parasanguinis*. Microbiology (2016) 162:376–83. 10.1099/mic.0.000226 26673783 PMC4766596

[24] Scoffield JA Duan D Zhu F Wu H. A commensal streptococcus hijacks a *Pseudomonas aeruginosa* exopolysaccharide to promote biofilm formation. PLoS Pathog. (2017) 13:e1006300. 10.1371/journal.ppat.1006300 28448633 PMC5407764

[25] Conrad D Haynes M Salamon P Rainey PB Youle M Rohwer F. Cystic fibrosis therapy: a community ecology perspective. Am J Respir Cell Mol Biol. (2013) 48:150–6. 10.1165/rcmb.2012-0059PS 23103995 PMC3604065

[26] Sherrard LJ Tunney MM Elborn JS. Antimicrobial resistance in the respiratory microbiota of people with cystic fibrosis. Lancet (2014) 384:703–13. 10.1016/S0140-6736(14)61137-5 25152272

[27] Kerem E Viviani L Zolin A MacNeill S Hatziagorou E Ellemunter H Factors associated with FEV1 decline in cystic fibrosis: analysis of the data of the ECFS patient registry. Eur Respir J. (2013) 43:125–33. 10.1183/09031936.00166412 23598952

[28] Goss CH Muhlebach MS. Review: *Staphylococcus aureus* and MRSA in cystic fibrosis. J Cyst Fibros. (2011) 10:298–306. 10.1016/j.jcf.2011.06.002 21719362

[29] Dalbøge CS Hansen CR Pressler T Høiby N Johansen HK. Chronic pulmonary infection with *Stenotrophomonas maltophilia* and lung function in patients with cystic fibrosis. J Cyst Fibros. (2011) 10:318–25. 10.1016/j.jcf.2011.03.006 21463972

[30] Costerton JW. Cystic fibrosis pathogenesis and the role of biofilms in persistent infection. Trends Microbiol. (2001) 9:50–2. 10.1016/S0966-842X(00)01918-1 11173226

[31] Heijerman H. Infection and inflammation in cystic fibrosis: a short review. J Cyst Fibros. (2005) 4 (Suppl. 2):3–5. 10.1016/j.jcf.2005.05.005 15970469

[32] Lyczak JB Cannon CL Pier GB. Lung infections associated with cystic fibrosis. Clin Microbiol Rev. (2002) 15:194–222. 10.1128/CMR.15.2.194-222.2002 11932230 PMC118069

[33] Armstrong D Grimwood K Carlin JB Carzino R Hull J Olinsky A. Severe viral respiratory infections in infants with cystic fibrosis. Pediatr Pulmonol. (1998) 26:371–9. 9888211 10.1002/(sici)1099-0496(199812)26:6<371::aid-ppul1>3.0.co;2-n

[34] Wat D Doull I. Respiratory virus infections in cystic fibrosis. Paediatr Respir Rev. (2003) 4:172–7. 10.1016/S1526-0542(03)00059-9 12880751

[35] Collinson J Nicholson KG Cancio E Ashman J Ireland DC Hammersley V. Effects of upper respiratory tract infections in patients with cystic fibrosis. Thorax (1996) 51:1115–22. 10.1136/thx.51.11.1115 8958895 PMC1090523

[36] Smyth AR Smyth RL Tong CY Hart CA Heaf DP. Effect of respiratory virus infections including rhinovirus on clinical status in cystic fibrosis. Arch Dis Child. (1995) 73:117–20. 10.1136/adc.73.2.117 7574853 PMC1511210

[37] Simoes EA. Respiratory syncytial virus infection. Lancet (1999) 354:847–52. 10.1016/S0140-6736(99)80040-3 10485741

[38] Abman SH Ogle JW Butler-Simon N Rumack CM Accurso FJ. Role of respiratory syncytial virus in early hospitalizations for respiratory distress of young infants with cystic fibrosis. J Pediatr. (1988) 113:826–30. 10.1016/S0022-3476(88)80008-8 3183835

[39] Rosenfeld M Emerson J Williams-Warren J Pepe M Smith A Montgomery AB. Defining a pulmonary exacerbation in cystic fibrosis. J Pediatr. (2001) 139:359–65. 10.1067/mpd.2001.117288 11562614

[40] Goss CH Burns JL Exacerbations in cystic fibrosis. 1: Epidemiology and pathogenesis. Thorax (2007) 62:360–7. 10.1136/thx.2006.060889 PMC2092469 17387214

[41] Smyth A Elborn JS. Exacerbations in cystic fibrosis: 3 management. Thorax (2008) 63:180–4. 10.1136/thx.2006.060905 18234661

[42] Hiatt PW Grace SC Kozinetz CA Raboudi SH Treece DG Taber LH. Effects of viral lower respiratory tract infection on lung function in infants with cystic fibrosis. Pediatrics (1999) 103:619–26. 10.1542/peds.103.3.619 10049966

[43] Wang EE Prober CG Manson B Corey M Levison H. Association of respiratory viral infections with pulmonary deterioration in patients with cystic fibrosis. N Engl J Med. (1984) 311:1653–8. 10.1056/NEJM198412273112602 6504106

[44] Johansen HK Høiby N. Seasonal onset of initial colonisation and chronic infection with *Pseudomonas aeruginosa* in patients with cystic fibrosis in Denmark. Thorax (1992) 47:109–11. 10.1136/thx.47.2.109 1549817 PMC463585

[45] van Ewijk BE van der Zalm MM Wolfs TFW van der Ent CK Viral respiratory infections in cystic fibrosis. J Cyst Fibros. (2005) 4:31–6. 10.1016/j.jcf.2005.05.011 15964785 PMC7105219

[46] van Ewijk BE van der Zalm MM Wolfs TFW Fleer A Kimpen JLL Wilbrink B Prevalence and impact of respiratory viral infections in young children with cystic fibrosis: prospective cohort study. Pediatrics (2008) 122:1171–6. 10.1542/peds.2007-3139 19047230

[47] Asner S Waters V Solomon M Yau Y Richardson SE Grasemann H. Role of respiratory viruses in pulmonary exacerbations in children with cystic fibrosis. J Cyst Fibros. (2012) 11:433–9. 10.1016/j.jcf.2012.04.006 22579414 PMC7105203

[48] Wark PAB Tooze M Cheese L Whitehead B Gibson PG Wark KF. Viral infections trigger exacerbations of cystic fibrosis in adults and children. Eur Respir J. (2012) 40:510–2. 10.1183/09031936.00202311 22855475

[49] Flight WG Bright-Thomas RJ Tilston P Mutton KJ Guiver M Morris J. Incidence and clinical impact of respiratory viruses in adults with cystic fibrosis. Thorax (2014) 69:247–53. 10.1136/thoraxjnl-2013-204000 24127019

[50] Goffard A Lambert V Salleron J Herwegh S Engelmann I Pinel C. Virus and cystic fibrosis: rhinoviruses are associated with exacerbations in adult patients. J Clin Virol. (2014) 60:147–53. 10.1016/j.jcv.2014.02.005 24637203 PMC7108260

[51] Schögler A Kopf BS Edwards MR Johnston SL Casaulta C Kieninger E. Novel antiviral properties of azithromycin in cystic fibrosis airway epithelial cells. Eur Respir J. (2015) 45:428–39. 10.1183/09031936.00102014 25359346

[52] Schögler A Stokes AB Casaulta C Regamey N Edwards MR Johnston SL. Interferon response of the cystic fibrosis bronchial epithelium to major and minor group rhinovirus infection. J Cyst Fibros. (2016) 15:332–9. 10.1016/j.jcf.2015.10.013 26613982 PMC7185532

[53] Kieninger E Singer F Tapparel C Alves MP Latzin P Tan H-L. High rhinovirus burden in lower airways of children with cystic fibrosis. Chest (2013) 143:782–90. 10.1378/chest.12-0954 23188200

[54] Dijkema JS van Ewijk BE Wilbrink B Wolfs TFW Kimpen JLL van der Ent CK. Frequency and duration of rhinovirus infections in children with cystic fibrosis and healthy controls: a longitudinal cohort study. Pediatr Infect Dis J. (2016) 35:379–83. 10.1097/INF.0000000000001014 26658528

[55] Rynda-Apple A Robinson KM Alcorn JF Influenza and bacterial super-infection: illuminating the immunologic mechanisms of disease. Infect Immun. (2015) 83:3764–70. 10.1128/IAI.00298-15 26216421 PMC4567631

[56] Shahangian A Chow EK Tian X Kang JR Ghaffari A Liu SY. Type I IFNs mediate development of postinfluenza bacterial pneumonia in mice. J Clin Invest. (2009) 119:1910–20. 10.1172/JCI35412 19487810 PMC2701856

[57] Lee B Robinson KM McHugh KJ Scheller EV Mandalapu S Chen C. Influenza-induced type I interferon enhances susceptibility to gram-negative and gram-positive bacterial pneumonia in mice. Am J Physiol Lung Cell Mol Physiol. (2015) 309:L158–67. 10.1152/ajplung.00338.2014 26001778 PMC4504975

[58] Robinson KM Choi S-M McHugh KJ Mandalapu S Enelow RI Kolls JK. Influenza A exacerbates *Staphylococcus aureus* pneumonia by attenuating IL-1β production in mice. J Immunol. (2013) 191:5153–9. 10.4049/jimmunol.1301237 24089191 PMC3827735

[59] Kudva A Scheller EV Robinson KM Crowe CR Choi S-M Slight SR. Influenza A inhibits Th17-mediated host defense against bacterial pneumonia in mice. J Immunol. (2011) 186:1666–74. 10.4049/jimmunol.1002194 21178015 PMC4275066

[60] McCullers JA. The co-pathogenesis of influenza viruses with bacteria in the lung. Nat Rev Microbiol. (2014) 12:252–62. 10.1038/nrmicro3231 24590244

[61] Sun K Metzger DW. Inhibition of pulmonary antibacterial defense by interferon-gamma during recovery from influenza infection. Nat Med. (2008) 14:558–64. 10.1038/nm1765 18438414

[62] Planet PJ Parker D Cohen TS Smith H Leon JD Ryan C. Lambda interferon restructures the nasal microbiome and increases susceptibility to *Staphylococcus aureus* superinfection. mBio (2016) 7:e01939–15. 10.1128/mBio.01939-15 26861017 PMC4752601

[63] Tarabichi Y Li K Hu S Nguyen C Wang X Elashoff D. The administration of intranasal live attenuated influenza vaccine induces changes in the nasal microbiota and nasal epithelium gene expression profiles. Microbiome (2015) 3:74. 10.1186/s40168-015-0133-2 26667497 PMC4678663

[64] Carmody LA Zhao J Schloss PD Petrosino JF Murray S Young VB. Changes in cystic fibrosis airway microbiota at pulmonary exacerbation. Ann Am Thorac Soc. (2013) 10:179–87. 10.1513/AnnalsATS.201211-107OC 23802813 PMC3960905

[65] Price KE Hampton TH Gifford AH Dolben EL Hogan DA Morrison HG. Unique microbial communities persist in individual cystic fibrosis patients throughout a clinical exacerbation. Microbiome (2013) 1:27. 10.1186/2049-2618-1-27 24451123 PMC3971630

[66] Stressmann FA Rogers GB Marsh P Lilley AK Daniels TWV Carroll MP. Does bacterial density in cystic fibrosis sputum increase prior to pulmonary exacerbation? J Cyst Fibros. (2011) 10:357–65. 10.1016/j.jcf.2011.05.002 21664196

[67] Chin M De Zoysa M Slinger R Gaudet E Vandemheen KL Chan F. Acute effects of viral respiratory tract infections on sputum bacterial density during CF pulmonary exacerbations. J Cyst Fibros. (2015) 14:482–9. 10.1016/j.jcf.2014.11.009 25544473 PMC7105172

[68] Esther CR Lin FC Kerr A Miller MB Gilligan PH. Respiratory viruses are associated with common respiratory pathogens in cystic fibrosis. Pediatr Pulmonol. (2014) 49:926–31. 10.1002/ppul.22917 24167159

[69] Miró-Cañís S Capilla-Rubio S Marzo-Checa L Fontanals-Aymerich D Sanfeliu-Sala I Espasa-Soley M. Multiplex PCR reveals that viruses are more frequent than bacteria in children with cystic fibrosis. J Clin Virol. (2017) 86:1–4. 10.1016/j.jcv.2016.11.004 27886635 PMC7106555

[70] Wat D Gelder C Hibbitts S Cafferty F Bowler I Pierrepoint M. The role of respiratory viruses in cystic fibrosis. J Cyst Fibros. (2008) 7:320–8. 10.1016/j.jcf.2007.12.002 18255355 PMC7105190

[71] Petersen NT Høiby N Mordhorst CH Lind K Flensborg EW Bruun B. Respiratory infections in cystic fibrosis patients caused by virus, chlamydia and mycoplasma–possible synergism with *Pseudomonas aeruginosa*. Acta Paediatr Scand. (1981) 70:623–8. 10.1111/j.1651-2227.1981.tb05757.x 6798822

[72] Gruenert DC Willems M Cassiman JJ Frizzell RA. Established cell lines used in cystic fibrosis research. J Cyst Fibros. (2004) 3 (Suppl 2):191–6. 10.1016/j.jcf.2004.05.040 15463957

[73] Randell SH Fulcher ML O'Neal W Olsen JC. Primary epithelial cell models for cystic fibrosis research. Methods Mol Biol. (2011) 742:285–310. 10.1007/978-1-61779-120-8_18 21547740

[74] Hendricks MR Lashua LP Fischer DK Flitter BA Eichinger KM Durbin JE. Respiratory syncytial virus infection enhances *Pseudomonas aeruginosa* biofilm growth through dysregulation of nutritional immunity. Proc Natl Acad Sci USA. (2016) 113:1642–7. 10.1073/pnas.1516979113 26729873 PMC4760822

[75] van Ewijk BE Wolfs TFW Aerts PC Van Kessel KPM Fleer A Kimpen JLL RSV mediates *Pseudomonas aeruginosa* binding to cystic fibrosis and normal epithelial cells. Pediatr Res. (2007) 61:398–403. 10.1203/pdr.0b013e3180332d1c 17515861

[76] Kiedrowski MR Gaston JR Kocak BR Coburn SL Lee S Pilewski JM *Staphylococcus* aureus biofilm growth on Cystic Fibrosis airway epithelial cells is enhanced during respiratory syncytial virus co-infection. mSphere (2018) 3:e00341–18. 10.1128/mSphere.00341-18 30111629 PMC6094059

[77] Hood MI Skaar EP. Nutritional immunity: transition metals at the pathogen-host interface. Nat Rev Microbiol. (2012) 10:525–37. 10.1038/nrmicro2836 22796883 PMC3875331

[78] Flo TH Smith KD Sato S Rodriguez DJ Holmes MA Strong RK. Lipocalin 2 mediates an innate immune response to bacterial infection by sequestrating iron. Nature (2004) 432:917–21. 10.1038/nature03104 15531878

[79] Bruce SR Atkins CL Colasurdo GN Alcorn JL. Respiratory syncytial virus infection alters surfactant protein A expression in human pulmonary epithelial cells by reducing translation efficiency. Am J Physiol Lung Cell Mol Physiol. (2009) 297:L559–67. 10.1152/ajplung.90507.2008 19525387 PMC2770795

[80] Griese M. Respiratory syncytial virus and pulmonary surfactant. Viral Immunol. (2002) 15:357–63. 10.1089/08828240260066279 12081017

[81] Numata M Chu HW Dakhama A Voelker DR. Pulmonary surfactant phosphatidylglycerol inhibits respiratory syncytial virus-induced inflammation and infection. Proc Natl Acad Sci USA. (2010) 107:320–5. 10.1073/pnas.0909361107 20080799 PMC2806703

[82] Siegel SJ Roche AM Weiser JN. Influenza promotes pneumococcal growth during coinfection by providing host sialylated substrates as a nutrient source. Cell Host Microbe (2014) 16:55–67. 10.1016/j.chom.2014.06.005 25011108 PMC4096718

[83] Baños-Lara MDR Piao B Guerrero-Plata A Differential mucin expression by respiratory syncytial virus and human metapneumovirus infection in human epithelial cells. Mediators Inflamm. (2015) 2015:347292 10.1155/2015/347292 25977598 PMC4421075

[84] Currier MG Lee S Stobart CC Hotard AL Villenave R Meng J. EGFR interacts with the fusion protein of respiratory syncytial virus strain 2–20 and mediates infection and mucin expression. PLoS Pathog (2016) 12:e1005622. 10.1371/journal.ppat.1005622 27152417 PMC4859522

[85] Aristoteli LP Willcox MDP. Mucin degradation mechanisms by distinct *Pseudomonas aeruginosa* isolates *in vitro*. Infect Immun. (2003) 71:5565–75. 10.1128/IAI.71.10.5565-5575.2003 14500475 PMC201046

[86] Flynn JM Niccum D Dunitz JM Hunter RC. Evidence and role for bacterial mucin degradation in cystic fibrosis airway disease. PLoS Pathog. (2016) 12:e1005846. 10.1371/journal.ppat.1005846 27548479 PMC4993466

[87] Son MS Matthews WJ Kang Y Nguyen DT Hoang TT. *In vivo* evidence of *Pseudomonas aeruginosa* nutrient acquisition and pathogenesis in the lungs of cystic fibrosis *patients*. Infect Immun. (2007). 75:5313–24. 10.1128/IAI.01807-06 17724070 PMC2168270

[88] Sun Z Kang Y Norris MH Troyer RM Son MS Schweizer HP. Blocking phosphatidylcholine utilization in *Pseudomonas aeruginosa*, via mutagenesis of fatty acid, glycerol and choline degradation pathways, confirms the importance of this nutrient source *in vivo*. PLoS ONE (2014) 9:e103778. 10.1371/journal.pone.0103778 25068317 PMC4113454

[89] Chattoraj SS Ganesan S Faris A Comstock A Lee WM Sajjan US *Pseudomonas aeruginosa* suppresses interferon response to rhinovirus infection in Cystic fibrosis, but not in normal bronchial epithelial cells. Infect Immun. (2011) 79:4131–45. 10.1128/IAI.05120-11 21825067 PMC3187241

[90] Dauletbaev N Das M Cammisano M Chen H Singh S Kooi C. Rhinovirus load is high despite preserved interferon-β response in cystic fibrosis bronchial epithelial cells. PLoS ONE (2015) 10:e0143129. 10.1371/journal.pone.0143129 26599098 PMC4658124

[91] Bomberger JM Ely KH Bangia N Ye S Green KA Green WR. *Pseudomonas aeruginosa* Cif protein enhances the ubiquitination and proteasomal degradation of the transporter associated with antigen processing (TAP) and reduces major histocompatibility complex (MHC) class I antigen presentation. J Biol Chem. (2014) 289:152–62. 10.1074/jbc.M113.459271 24247241 PMC3879540

[92] Høiby N Bjarnsholt T Givskov M Molin S Ciofu O. Antibiotic resistance of bacterial biofilms. Int J Antimicrob Agents (2010) 35:322–32. 10.1016/j.ijantimicag.2009.12.011 20149602

[93] Lambert PA. Mechanisms of antibiotic resistance in *Pseudomonas aeruginosa*. J R Soc Med. (2002) 95 (Suppl. 41):22–6. 12216271 PMC1308633

[94] Melvin JA Lashua LP Kiedrowski MR Yang G Deslouches B Montelaro RC. Simultaneous antibiofilm and antiviral activities of an engineered antimicrobial peptide during virus-bacterium coinfection. mSphere (2016) 1:e00083–16. 10.1128/mSphere.00083-16 27303744 PMC4888888

[95] Champney WS Burdine R. Macrolide antibiotics inhibit 50S ribosomal subunit assembly in *Bacillus subtilis* and *Staphylococcus aureus*. Antimicrob Agents Chemother. (1995) 39:2141–4. 10.1128/AAC.39.9.2141 8540733 PMC162898

[96] Amsden GW. Anti-inflammatory effects of macrolides–an underappreciated benefit in the treatment of community-acquired respiratory tract infections and chronic inflammatory pulmonary conditions? J Antimicrob Chemother. (2005) 55:10–21. 10.1093/jac/dkh519 15590715

[97] Gielen V Johnston SL Edwards MR. Azithromycin induces anti-viral responses in bronchial epithelial cells. Eur Respir J. (2010) 36:646–54. 10.1183/09031936.00095809 20150207

[98] Suzuki T Yamaya M Sekizawa K Hosoda M Yamada N Ishizuka S Bafilomycin A1 inhibits rhinovirus infection in human airway epithelium: effects on endosome and ICAM-1. Am J Physiol Lung Cell Mol Physiol. (2001) 280:L1115–27. 10.1152/ajplung.2001.280.6.L1115 11350790

[99] Jang YJ Kwon H-J Lee B-J. Effect of clarithromycin on rhinovirus-16 infection in A549 cells. Eur Respir J. (2006) 27:12–9. 10.1183/09031936.06.00008005 16387930

[100] Phaff SJ Tiddens HAWM Verbrugh HA Ott A. Macrolide resistance of *Staphylococcus aureus* and *Haemophilus* species associated with long-term azithromycin use in cystic fibrosis. J Antimicrob Chemother. (2006) 57:741–6. 10.1093/jac/dkl014 16469851

[101] Groothuis JR Simoes EA Levin MJ Hall CB Long CE Rodriguez WJ. Prophylactic administration of respiratory syncytial virus immune globulin to high-risk infants and young children. The respiratory syncytial virus immune globulin study group. N Engl J Med. (1993) 329:1524–30. 10.1056/NEJM199311183292102 8413475

[102] Null DM Bimle C Weisman LE Johnson K Steichen J Singh S Palivizumab, a humanized respiratory syncytial virus monoclonal antibody, reduces hospitalization from respiratory syncytial virus infection in high-risk infants. Pediatrics (1998) 102:531–7. 10.1542/peds.102.3.531 9724660

[103] Giebels K Marcotte JE Podoba J Rousseau C Denis MH Fauvel V. Prophylaxis against respiratory syncytial virus in young children with cystic fibrosis. Pediatr Pulmonol. (2008) 43:169–74. 10.1002/ppul.20751 18085710

[104] Bjornson C Chan P Li A Paes B Lanctôt KL Mitchell I. Palivizumab prophylaxis for respiratory syncytial virus in infants with cystic fibrosis: is there a need? Eur J Clin Microbiol Infect Dis. (2018) 37:1113–8. 10.1007/s10096-018-3225-7 29557081

[105] Linnane B Kiernan MG O'Connell NH Kearse L Dunne CP. Anti-RSV prophylaxis efficacy for infants and young children with cystic fibrosis in Ireland. Multidiscip Respir Med. (2015) 10:32. 10.1186/s40248-015-0029-9 26473032 PMC4607252

[106] McGirr AA Schwartz KL Allen U Solomon M Sander B The cost-effectiveness of palivizumab in infants with cystic fibrosis in the Canadian setting: a decision analysis model *Hum Vaccin Immunother*. (2017) 13:599–606. 10.1080/21645515.2016.1235670 PMC5360124 27768505

[107] Buchs C Dalphin M-L Sanchez S Perceval M Coutier L Mainguy C Palivizumab prophylaxis in infants with cystic fibrosis does not delay first isolation of *Pseudomonas aeruginosa* or *Staphylococcus aureus*. Eur J Pediatr. (2017) 176:891–7. 10.1007/s00431-017-2926-8 28508992

[108] Groves HE Jenkins L Macfarlane M Reid A Lynn F Shields MD. Efficacy and long-term outcomes of palivizumab prophylaxis to prevent respiratory syncytial virus infection in infants with cystic fibrosis in Northern Ireland. Pediatr Pulmonol. (2016) 51:379–85. 10.1002/ppul.23376 26808981

[109] Wainwright CE Elborn JS Ramsey BW Marigowda G Huang X Cipolli M Lumacaftor-ivacaftor in patients with cystic fibrosis homozygous for Phe508del CFTR. N Engl J Med. (2015) 373:220–31. 10.1056/NEJMoa1409547 25981758 PMC4764353

[110] Taylor-Cousar JL Munck A McKone EF van der Ent CK Moeller A Simard C. Tezacaftor-ivacaftor in patients with cystic fibrosis homozygous for Phe508del. N Engl J Med. (2017) 377:2013–23. 10.1056/NEJMoa1709846 29099344

[111] Boyle MP Bell SC Konstan MW McColley SA Rowe SM Rietschel E. A CFTR corrector (lumacaftor) and a CFTR potentiator (ivacaftor) for treatment of patients with cystic fibrosis who have a phe508del CFTR mutation: a phase 2 randomised controlled trial. Lancet Respir Med. (2014) 2:527–38. 10.1016/S2213-2600(14)70132-8 24973281

[112] Clancy JP Rowe SM Accurso FJ Aitken ML Amin RS Ashlock MA. Results of a phase IIa study of VX-809, an investigational CFTR corrector compound, in subjects with cystic fibrosis homozygous for the F508del-CFTR mutation. Thorax (2012) 67:12–8. 10.1136/thoraxjnl-2011-200393 21825083 PMC3746507

[113] Ratjen F Hug C Marigowda G Tian S Huang X Stanojevic S. Efficacy and safety of lumacaftor and ivacaftor in patients aged 6-11 years with cystic fibrosis homozygous for F508del-CFTR: a randomised, placebo-controlled phase 3 trial. Lancet Respir Med. (2017) 5:557–67. 10.1016/S2213-2600(17)30215-1 28606620

[114] Reznikov LR Abou Alaiwa MH Dohrn CL Gansemer ND Diekema DJ Stoltz DA. Antibacterial properties of the CFTR potentiator ivacaftor. J Cyst Fibros. (2014) 13:515–9. 10.1016/j.jcf.2014.02.004 24618508 PMC4718582

[115] Bernarde C Keravec M Mounier J Gouriou S Rault G Férec C. Impact of the CFTR-potentiator ivacaftor on airway microbiota in cystic fibrosis patients carrying a G551D Mutation. PLoS ONE (2015) 10:e0124124. 10.1371/journal.pone.0124124 25853698 PMC4390299

[116] Hisert KB Heltshe SL Pope C Jorth P Wu X Edwards RM. Restoring cystic fibrosis transmembrane conductance regulator function reduces airway bacteria and inflammation in people with cystic fibrosis and chronic lung infections. Am J Respir Crit Care Med. (2017) 195:1617–28. 10.1164/rccm.201609-1954OC 28222269 PMC5476912

[117] Hisert KB Schoenfelt KQ Cooke G Grogan B Launspach JL Gallagher CG. Ivacaftor-induced proteomic changes suggest monocyte defects may contribute to the pathogenesis of cystic fibrosis. Am J Respir Cell Mol Biol. (2016) 54:594–7. 10.1165/rcmb.2015-0322LE 27035067 PMC4821059

[118] Willner D Furlan M Haynes M Schmieder R Angly FE Silva J. Metagenomic analysis of respiratory tract DNA viral communities in cystic fibrosis and non-cystic fibrosis individuals. PLoS ONE (2009) 4:e7370. 10.1371/journal.pone.0007370 19816605 PMC2756586

[119] Lim YW Schmieder R Haynes M Willner D Furlan M Youle M. Metagenomics and metatranscriptomics: windows on CF-associated viral and microbial communities. J Cyst Fibros. (2013) 12:154–64. 10.1016/j.jcf.2012.07.009 22951208 PMC3534838

[120] Secor PR Sweere JM Michaels LA Malkovskiy AV Lazzareschi D Katznelson E. Filamentous bacteriophage promote biofilm assembly and function. Cell Host Microbe (2015) 18:549–59. 10.1016/j.chom.2015.10.013 26567508 PMC4653043

[121] Manrique P Bolduc B Walk ST van der Oost J de Vos WM Young MJ. Healthy human gut phageome. Proc Natl Acad Sci USA. (2016) 113:10400–5. 10.1073/pnas.1601060113 27573828 PMC5027468

[122] Manrique P Dills M Young M. The human gut phage community and its implications for health and disease. Viruses (2017) 9:141. 10.3390/v9060141 28594392 PMC5490818

[123] Damron FH Oglesby-Sherrouse AG Wilks A Barbier M. Dual-seq transcriptomics reveals the battle for iron during *Pseudomonas aeruginosa* acute murine pneumonia. Sci Rep. (2016) 6:39172. 10.1038/srep39172 27982111 PMC5159919

[124] Zhou B Xiao JF Tuli L Ressom HW. LC-MS-based metabolomics. Mol BioSyst. (2012) 8:470–81. 10.1039/C1MB05350G 22041788 PMC3699692

[125] Gilchrist FJ Bright-Thomas RJ Jones AM Smith D Španěl P Webb AK. Hydrogen cyanide concentrations in the breath of adult cystic fibrosis patients with and without *Pseudomonas aeruginosa* infection. J Breath Res. (2013) 7:026010. 10.1088/1752-7155/7/2/026010 23680696

[126] Gilchrist FJ Belcher J Jones AM Smith D Smyth AR Southern KW. Exhaled breath hydrogen cyanide as a marker of early *Pseudomonas aeruginosa* infection in children with cystic fibrosis. ERJ Open Res. (2015) 1:00044–2015. 10.1183/23120541.00044-2015 27730156 PMC5005121

[127] Pabary R Huang J Kumar S Alton EWFW Bush A Hanna GB. Does mass spectrometric breath analysis detect *Pseudomonas aeruginosa* in cystic fibrosis? Eur Respir J. (2016) 47:994–7. 10.1183/13993003.00944-2015 26846826

[128] Bos LDJ Meinardi S Blake D Whiteson K. Bacteria in the airways of patients with cystic fibrosis are genetically capable of producing VOCs in breath. J Breath Res. (2016) 10:047103. 10.1088/1752-7163/10/4/047103 27991430

[129] Purcaro G Rees CA Melvin JA Bomberger JM Hill JE. Volatile fingerprinting of *Pseudomonas aeruginosa* and respiratory syncytial virus infection in an *in vitro* cystic fibrosis co-infection model. J Breath Res. (2018) 12:046001. 10.1088/1752-7163/aac2f1 29735804 PMC6057612

